# Indonesia: An Emerging Market Economy Beset by Neglected Tropical Diseases (NTDs)

**DOI:** 10.1371/journal.pntd.0002449

**Published:** 2014-02-27

**Authors:** Melody Tan, Rita Kusriastuti, Lorenzo Savioli, Peter J. Hotez

**Affiliations:** 1 Department of Bioengineering, Rice University, Houston, Texas, United States of America; 2 Vector Borne and Neglected Tropical Diseases Unit, South-East Asia Regional Organization (SEARO), World Health Organization, New Delhi, India; 3 Department of Neglected Tropical Diseases, World Health Organization (WHO), Geneva, Switzerland; 4 Departments of Pediatrics and Microbiology and Molecular Virology, National School of Tropical Medicine, Baylor College of Medicine, Houston, Texas, United States of America; 5 Sabin Vaccine Institute and Texas Children's Hospital Center for Vaccine Development, Houston, Texas, United States of America; 6 James A. Baker III Institute for Public Policy, Rice University, Houston, Texas, United States of America

*Despite an enormous population and growing economy, the nation of Indonesia has some of the world's highest concentrations of neglected tropical diseases (NTDs). These NTDs may thwart future national growth and recent gains. Yet, Indonesia and its Ministry of Health, together with the World Health Organization (WHO), have embarked on an ambitious effort to quickly assemble a health and scientific infrastructure suitable for eliminating its NTDs.*

The nation of Indonesia is the world's largest island nation (approximately 17,000 islands of which 5,000–6,000 are inhabited) and the fourth most populated nation behind China, India, and the United States (Figure 1) [1]. The nation is tied with the Netherlands as the world's 16th largest economy [1]. According to the World Bank, Indonesia together with South Korea and the BRIC (Brazil, Russia, India, and China) countries will account for more than half of the world's economic growth by 2025 [2].

**Figure 1:**
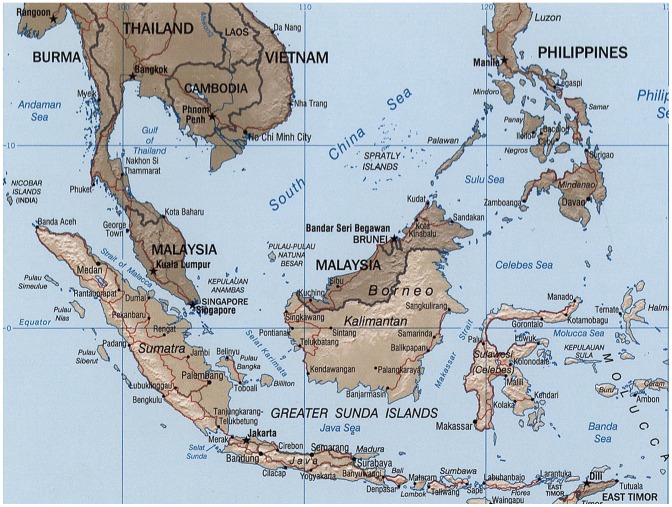
Map of Indonesia. From Wikimedia Commons. Available: http://commons.wikimedia.org/wiki/File:Indonesia_2002_CIA_map.png. Accessed 6 June 2013.

Despite the future promise of these economic gains, Indonesia is also simultaneously hobbled by a staggering level of extreme poverty. According to the World Bank, of Indonesia's estimated population of 242 million [3], an estimated 46% (approximately 111 million people) live on less than $2 per day [4], while 18% (44 million people) live on less than $1.25 per day [5].

Today, one of the most potent forces that currently traps Indonesia's poorest 111 million people in poverty and could eventually threaten Indonesia's economic potential is a group of NTDs affecting the region. The NTDs are chronic conditions with clinical features similar to many noncommunicable diseases [6]. When they become widespread [6], these diseases have the ability to thwart or stall economies because of their adverse impact on child development, labor, and the health of girls and women [7].

Indeed, Indonesia's “bottom 111” million suffer from an extraordinary level of NTDs, led by widespread helminth infections, such as soil-transmitted helminth (STH) infections and lymphatic filariasis (LF), and neglected bacterial infections, such as yaws and leptospirosis (Table 1) [8]. Moreover, Indonesia is the only country in the WHO's South-East Asia Region with endemic schistosomiasis [8] (the other Asian countries with endemic schistosomiasis are in WHO's Western Pacific region), and the nation is facing a serious and emerging threat from dengue fever [9].

**Table 1:** The major neglected tropical diseases of Indonesia.

Disease	Number of Cases or People at Risk	Percentage of Global Disease Burden or Population at Risk	References	Additional Comments
Trichuriasis	95 million	16%	[8], [10]–[12]	STH infections in 31 of 33 provinces in Indonesia^1^
Ascariasis	90 million	11%	[8], [10]–[12]	STH infections in 31 of 33 provinces in Indonesia^1^
Hookworm	62 million	11%	[8], [10]–[12]	STH infections in 31 of 33 provinces in Indonesia^1^
Schistosomiasis	25,000–50,000 at risk	<1%	[8], [13]	All cases in Central Sulawesi Province
Lymphatic Filariasis	125 million at risk	9%	[8], [14]–[16]	Highest prevalence in Eastern Indonesia
Leprosy	20,023 new cases in 2011	9% of new global cases in 2011	[8], [17]	Especially in Central and West Java
Yaws	8,039 cases reported in 2009	Not determined	[8]	Highly endemic in Papua, Southeast Sulawesi, and Nusa Tenggara Timur provinces
Leptospirosis	Not determined	Not determined	[19]	
Dengue	3,436 deaths	58% of the deaths in South-East Asia 23% of deaths globally	[9], [20]	Indonesia has the second largest number of cases worldwide
Chikungunya and Japanese encephalitis	Not determined	Not determined	[21], [22]	

1 Only STH infections in general are specified.

## STH Infections

Based on estimates published a decade ago, Indonesia may now rank only behind India in terms of the total number of STH infections affecting its population, including 95 million, 90 million, and 62 million people with trichuriasis, ascariasis, and hookworm infection, respectively [10]. Based on the 2006 estimates for the total number of these helminth infections globally [11], between 11 and 16% of the world's cases of STH infections occur in Indonesia alone. According to the Indonesian Ministry of Health (IMOH), most of Indonesia's population—approximately 195 million people—live in STH-endemic areas (31 of Indonesia's 33 provinces), including 13 million preschool children and 37 million school-age children who should be targeted for annual or twice-yearly anthelminthic therapy with either albendazole or mebendazole [8]. In 2009, roughly 10% of these children received anthelminthic therapy as part of the albendazole component of their LF mass annual treatments (diethylcarbamazine [DEC] and albendazole combination therapy) [8]. However, there is a need to significantly expand regular and periodic deworming with either albendazole or mebendazole, especially for a significant number of school-age children (13.5 million) and preschool-age children (4.6 million) who live in STH-endemic areas where LF is not endemic [8]. Some of these children are being targeted for anthelminthic treatment through the World Food Programme (WFP) [8]. Ultimately, the goal of the IMOH is to treat all children at risk for STH infections as per the guidelines of the 2001 World Health Assembly resolution on deworming, and implement this treatment together with programs of health education and possibly behavioral modification [8], [12]. As described below, as a component of its NTD National Action Plan (2011–2015), the Indonesian Ministry of Health has been conducting mass drug administration (MDA) for STH infections in collaboration with the Directorates of Maternal-Child Health (MCH) and Nutrition, local nongovernmental organizations (NGOs) (“Kusuma Buana”), and with technical and financial assistance from RTI International and the U.S. Agency for International Development (USAID). In addition, the IMOH's Centre for Health Promotion has implemented a program for hygiene education and outreach [8] that addresses defecation and hand-washing practices.

## Schistosomiasis

Schistosomiasis, caused by *Schistosoma japonicum*, is mostly confined to two or three isolated areas—mostly in Lindu Valley and Napu Valley—located in the province of Central Sulawesi [8], [13]. According to the IMOH, there are approximately 25,000–50,000 people at risk for acquiring *S. japonicum* infection [8], which represents less than 1% of the people at risk for schistosomiasis globally. A significant hurdle for the control and elimination of schistosomiasis in Central Sulawesi has been ongoing transmission of infection from animals (including cows, buffaloes, sheep, goats, and other animals), similar to *S. japonicum* infections in China and the Philippines. A detailed plan is being developed by the IMOH to eliminate schistosomiasis in Central Sulawesi by 2020 through praziquantel MDA of the entire population over the age of four, together with health education, environmental management, and agro-engineering [8].

## Lymphatic Filariasis

**Figure 2:**
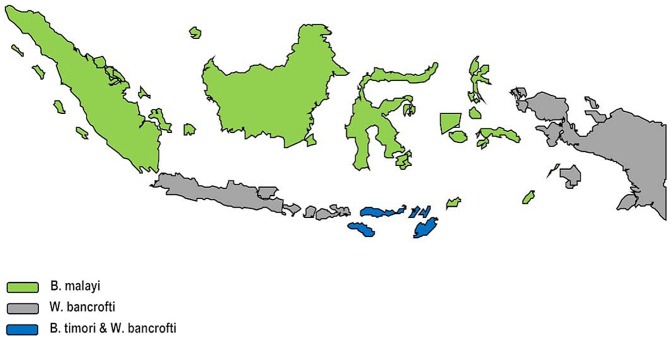
Geographic distribution of the three major filarial species in Indonesia. Data from Indonesian Ministry of Health. Map showing distribution of *B. malayi* (Sumatra, Kalimantan, Sulawesi), *W. bancrofti* (Java, Bali, Nusa Tenggara Barat [NTB] and Papua), and *B. malayi* and *B. timori* in Nusa Tenggara Timur (NTT).

Like the three major STH infections, LF is also widespread in Indonesia [8], [14]–[16]. According to the IMOH, approximately 125 million Indonesians are at risk for acquiring LF (representing approximately 9% of the global population at risk for LF [16]), with the highest prevalence rates in Maluku, Papua, West Irian Jaya, East Nusa Tenggara, and North Maluku provinces in Eastern Indonesia [8]. The LF situation in Indonesia is unusual in that all three species, i.e., *Brugia malayi*, *Brugia timori*, and *Wuchereria bancrofti*, are endemic, with most of the infections caused by *B. malayi* (Figure 2) [8]. The major effort to control and eliminate LF in Indonesia is mass drug administration with DEC and albendazole. However, the current drug coverage in the major endemic districts is only approximately 30% [8]. Among the factors that have thwarted better access to DEC and albendazole are the social stigmatization linked to LF [14], the vast island geography and challenge of reaching remote populations, and, in some cases, compliance issues in terms of persuading the at-risk populations to take their antifilarial medication [15]. Another obstacle has been inconsistent budget allocations for LF elimination in association with decentralization of government services. Moreover, the immunochromatographic test (ICT) card used for detecting parasite antigens in LF infections caused by *W. bancrofti* does not work as well in areas where *B. malayi* and *B. timori* are widely prevalent. The goals of the IMOH LF control program are to achieve LF elimination by 2020 through an ambitious program of annual mass drug administration and to reach at least 65% of the population at risk by 2016 [8]. Currently, GlaxoSmithKline is providing albendazole free of charge (sent through WHO), while DEC is purchased locally with the national budget [8].

## Neglected Bacterial Infections

The major neglected bacterial infections in Indonesia are leprosy, yaws, leptospirosis, and rickettsia infections. Almost 10% of the world's new cases of leprosy now occur in Indonesia, with approximately 20,000 new cases in 2011 [17]. According to the IMOH, a significant number of cases exhibit grade 2 disability. Thus, although leprosy has been eliminated at the national level (as defined by less than one case per 10,000 people), there are several provinces, especially in Java (East, Central, and West Java), where, locally, those elimination targets have not yet been met [8]. Therefore, there are concentrated efforts underway to increase access to multidrug therapy, using donated drugs from the Novartis Foundation together with operational and technical assistance to the IMOH from Netherlands Leprosy Relief and the Sasakawa Foundation [8]. Yaws also remains endemic, with five provinces exhibiting high disease burden, especially in Papua, Southeast Sulawesi, and Nusa Tenggara Timur province [8]. The current approach has been mass annual treatment with benzathine penicillin, especially in areas where LF mass drug administration is being conducted [8], although it is unclear if this approach might consider annual treatment with azithromycin as an alternative, as has been proposed for Papua New Guinea [18]. Finally, leptospirosis and rickettsia infections are also believed to be widespread [19], but there are no disease burden data for these two infections. With regard to the former, in 2007 and 2009 as well as in 2010 and 2011 Indonesia experienced an outbreak of leptospirosis, mostly in the Central Java and Jogyakarta areas, where it was noted to occur in association with Mount Merapi eruptions as well as in paddy field areas, forest and flood areas; most of the affected individuals were farmers and forest workers. The Zoonosis subdirectorate of IMOH implemented a control program which included case detection and treatment, rodent control, health education to the community, and working intersectorally with agriculture and veterinary sectors.

## Neglected Viral Infections

Beginning in 1968, dengue emerged in Indonesia where it has remained a major public health threat. Today, all four serotypes are present, and the number of dengue cases reported annually to the WHO ranks Indonesia as having the second largest number of cases worldwide. Shepard et al. have recently estimated the disease burden and economic impact of dengue in South-East Asia [9]. They determined that more than half of the almost 6,000 deaths that occur annually in the region occur in Indonesia, and almost half of the region's hospitalizations [9]. Thus, Indonesia alone may be responsible for almost a quarter of all global deaths from dengue [20]. Moreover, the costs of dengue exceed $300 million annually in Indonesia, a figure that represents just over a third of the economic burden of dengue in the region [9]. Beginning in 2009, the incidence of dengue fever and dengue hemorrhagic fever began to decrease in association with enhanced public health control measures, which included community mobilization with weekly clearing of mosquito breeding sites (and a program of cleaning, drying, covering, recycling, and larviciding). Dengue control has been given the highest level of political support by the Indonesian President, while the Association of Southeast Asian Nations (ASEAN) together with the WHO has inaugurated Dengue Day to reiterate the need for all sectors of society to unite against this disease [21].

Chikungunya and Japanese encephalitis are also found in Indonesia [22], [23], although no disease burden or economic estimates for these diseases are available. For Chikungunya control the IMOH developed a prevention and control program similar to its dengue activities, while for Japanese encephalitis a vaccine is available.

## Approaches to Disease Control and Elimination

The IMOH has drafted an ambitious roadmap for NTD control and elimination, with an emphasis on STH control, LF and schistosomiasis elimination, and leprosy and yaws elimination [8]. A cornerstone for this “Plan of Action” is to follow WHO guidelines and algorithms based on areas where people are at risk for coinfections with STH infections, LF, and schistosomiasis [8]. Included are the 125 million people at risk for LF who will also receive albendazole for their STHs, an additional 13.5 million school-age children who require treatment for STH infections but who do not live in LF-endemic areas, 25,000–50,000 individuals who may also require praziquantel for schistosomiasis, and elimination strategies for leprosy and yaws [8]. These national efforts in Indonesia represent a key element for larger WHO elimination efforts for LF, leprosy, and yaws in the South-East Asia Region [24]. The IMOH also recognizes the need to integrate these efforts with improvements in water and sanitation and has also implemented plans for integrated vector management, which includes linking NTD chemotherapy with water, sanitation, and health education efforts [8].

Indonesia has been designated for financial support for its NTDs from the USAID [25], and, along with the commitment of funds from the IMOH as specified in its national plan for NTD control and elimination, the added USAID should go a long way in helping the IMOH achieve its NTD goals and targets. The Australian Department of Foreign Affairs and Trade and UNICEF are also committed to poverty reduction, NTD control, and measures for clean water and sanitation in Indonesia [8], [26], [27]. In parallel, Indonesia's major research institutes and organizations, including its major vaccine enterprise Biofarma, the Eijkman Institute of Molecular Biology, the National Institute of Health and Research Development, and the University of Indonesia, have a major role in helping to shape and develop new diagnostics, drugs, and vaccines for NTDs affecting the region. Of note is a new joint venture between the Eijkman Institute and Sanofi Pasteur to target dengue [28].

There is no question that unless Indonesia and its IMOH take action, the highly prevalent NTDs will thwart national aspirations for sustained economic growth. However, the IMOH, in concert with the WHO and key academic and research institute partners, is poised to launch an ambitious assault on the NTDs, which represents a major step towards poverty reduction and elimination in South-East Asia.
